# Age-period-cohort analysis of schizophrenia incidence in China and Japan from 1990 to 2021: long-term trends and risk changes

**DOI:** 10.3389/fpsyt.2025.1626821

**Published:** 2025-12-02

**Authors:** Yifan Hao, Hu Zhao, Zhixian Xu, Yu Feng, Ruhai Bai, Hui Gu

**Affiliations:** 1 School of Marxism, Nanjing University of Science & Technology, Nanjing, China; 2 School of Public Affairs, Nanjing University of Science & Technology, Nanjing, China

**Keywords:** schizophrenia, age-period-cohort models, China, Japan, incidence

## Abstract

**Background:**

Schizophrenia represents the psychiatric disorder with the highest per capita social cost globally, with East Asia bearing a disproportionate burden of schizophrenia episodes and associated disability-adjusted life years. This study aimed to comprehensively evaluate the long-term trends in schizophrenia incidence in China and Japan from 1990 to 2021.

**Methods:**

Data were extracted from the Global Burden of Disease 2021 study and systematically analyzed using age-period-cohort modeling approaches to disentangle temporal trends.

**Results:**

The overall drift values for schizophrenia incidence were -0.25% (95% CI: -0.28% to 0.23%) in China and 0.30% (95% CI: 0.20% to 0.41%) in Japan. In China, local drift values were positive only in the 10–25 age group and negative across all other age groups. For Japanese males, local drift values were negative except in the 15–25 age group. The longitudinal age curves for schizophrenia in the both countries demonstrated a characteristic inverted “V” shape, with peak incidence occurring in the 20–24 age group. Regarding period effects, China showed an overall decreasing trend that reversed toward unfavorable outcomes after 2014, while Japan also exhibited a decreasing trend recently, albeit with greater fluctuations. The cohort effect in China displayed a U-shaped pattern, whereas Japan showed an overall upward trend with a slight midpoint decrease, in the middle, followed by a sharp rise in risk for cohorts born after 2000.

**Conclusion:**

Despite established mental health initiatives in both China and Japan, schizophrenia risk is increasing among youth cohorts in both countries. This finding underscores the urgent need for both nations to develop more effective, demographically targeted approached to improve mental health outcomes across different population segments.

## 1 Introduction

Schizophrenia is a severe mental disorder characterized by a diverse spectrum of symptoms, including delusions, hallucinations, disorganized speech, extremely disorganized or psychotic behavior, and diminished emotional expression or demoralization (1). As the psychiatric condition with highest per capita social cost, individuals with schizophrenia exhibit risks for developing comorbid somatic conditions, including HIV infection, obstetric complications, and cardiovascular disease (2, 3). Among all mental disorders, schizophrenia ranks third in terms of disability-adjusted life years (DALYs), preceded only by depression and anxiety disorders (4). Notably, the disease burden attributed to schizophrenia has not diminished but has instead increased steadily over the past three decades (5).

In the global context, East Asia bears a disproportionate burden of schizophrenia, reporting the highest absolute number of schizophrenia episodes and corresponding DALYs worldwide, while maintaining the third-highest regional prevalence of this disorder (4, 6). Between 1990 and 2019, China’s age-standardized incidence of schizophrenia demonstrated an upward trajectory, aligning with global trends (7). Within East Asia, China and Japan represent contrasting socioeconomic paradigms as developing and developed nations, respectively, with substantial differences in economic development, healthcare resource allocation, and socio-cultural frameworks. Nevertheless, as neighboring countries, they share significant genetic and cultural foundations, particularly regarding cognitive patterns, emotional expression, and response to mental disorders (8). Furthermore, China is currently undergoing rapid urbanization and economic transformation, processes that profoundly impact population mental health, while Japan, despite possessing a relatively advanced mental health service infrastructure, continues to confront significant challenges in addressing serious psychiatric conditions (9).

Although previous research has examined trends in schizophrenia disease burden at global, national, and regional levels, analyzed incidence rate variations across countries with differing socioeconomic development, and independently investigated temporal dynamics of schizophrenia burden in China, comprehensive comparative analyses of schizophrenia incidence trends within the East Asian region remain conspicuously absent (6, 7, 10). To address this research gap, the present study provides an in-depth comparative analysis of schizophrenia incidence trends between China and Japan, utilizing data from the Global Burden of Disease Study (GBD) 2021 and applying age-period-cohort modeling methodology. This approach enables the identification of distinct risk factors influencing schizophrenia incidence in each country and facilitates the development of targeted public health strategies customized to address country-specific needs.

## 2 Methodology

### 2.1 Data sources

Data for this study were obtained from the Global Burden of Disease (GBD) 2021 database, which provides comprehensive and internally consistent estimates for 371 diseases and injuries across 204 countries and territories from 1990 to 2021. For our analysis, we extracted schizophrenia incidence and demographic data from the Institute for Health Metrics and Evaluation’s website (https://vizhub.healthdata.org/gbd-results/) for individuals aged 10–69 years in China and Japan spanning the period 1990 to 2021. In this study, schizophrenia was defined as a chronic psychotic disorder characterized by positive symptoms (including delusions, hallucinations, and thought triggers) and negative symptoms (such as emotional flatness, diminished interest, and emotional withdrawal). The GBD disease modeling included cases meeting the Diagnostic and Statistical Manual of Schizophrenia (DSM) or International Classification of Diseases (ICD) diagnostic criteria for schizophrenia (DSM-IV-TR: 295.10-295.30, 295.60, 295.90; ICD-10: F20). Multiple versions of these diagnostic systems were accepeted (11).

### 2.2 Data analysis

The age-period-cohort (APC) model, widely employed in epidemiological research to analyze trends in disease morbidity or mortality, was utilized to decompose the overall temporal trends into distinct age, period, and cohort effects. The APC model can be generally expressed as:


$$Y=log(\text{M})=u+\text{α}Age_{i}+\text{β}Period_{j}+\text{γ}Cohort_{k}+\epsilon$$

where M represents the incidence rate of the corresponding age group, μ is the intercept term, α, β, and γ denote the age, period, and cohort effects respectively, and ϵ is the random error. To address the perfect collinearity among age, period, and cohort variables, weighted least squares regression was implemented to effectively delineate these effects (11).

In the APC model framework, the age effect captures changes in schizophrenia incidence attributable to age-related factors, reflecting the impact of biological or sociological developmental processes. The period effect reflects risk variations between specific time points, while the cohort effect represents overall risk changes across successive birth cohorts. Our analysis estimated five key parameters to quantify theses three effects: (1) local drift and (2) net drift, representing the expected age-adjusted and age-specific annual percentage changes over time, respectively; (3) the longitudinal age curve, which illustrates the age effect by depicting the expected age-specific incidence of schizophrenia at the reference cohort point adjusted for the period effects; and (4) period ratio and (5) cohort ratio, representing the period and cohort effects as the ratio of age-specific schizophrenia rates to the reference period or cohort incidence rates.

For analytical purposes, we stratified 1992–2021 observation timeframe into six 5-year periods and divided the observation cohort (ages 10–69 years) into 12 age bands at 5-year intervals, yielding 17 consecutive birth cohorts spanning from 1925-1929 (median 1927) to 2005-2009 (median 2007). All parameters were estimated using the National Cancer Institute APC web tool (Biostatistics Branch, National Cancer Institute, Bethesda, Maryland, USA), which by default utilizes the median values of age groups, period groups, and cohort groups as reference points. In this study, the central age group (35–39 years), period (2002-2006), and birth cohort (1965–1969 cohort) were designated as reference categories. All statistical tests were two-sided with a significance threshold of 5%. Results were evaluated using the chi-square test, with p-values less than 0.05 considered statistically significant.

## 3 Results

### 3.1 Trends in schizophrenia incidence rates in China and Japan from 1990 to 2021

**Figure 1:**
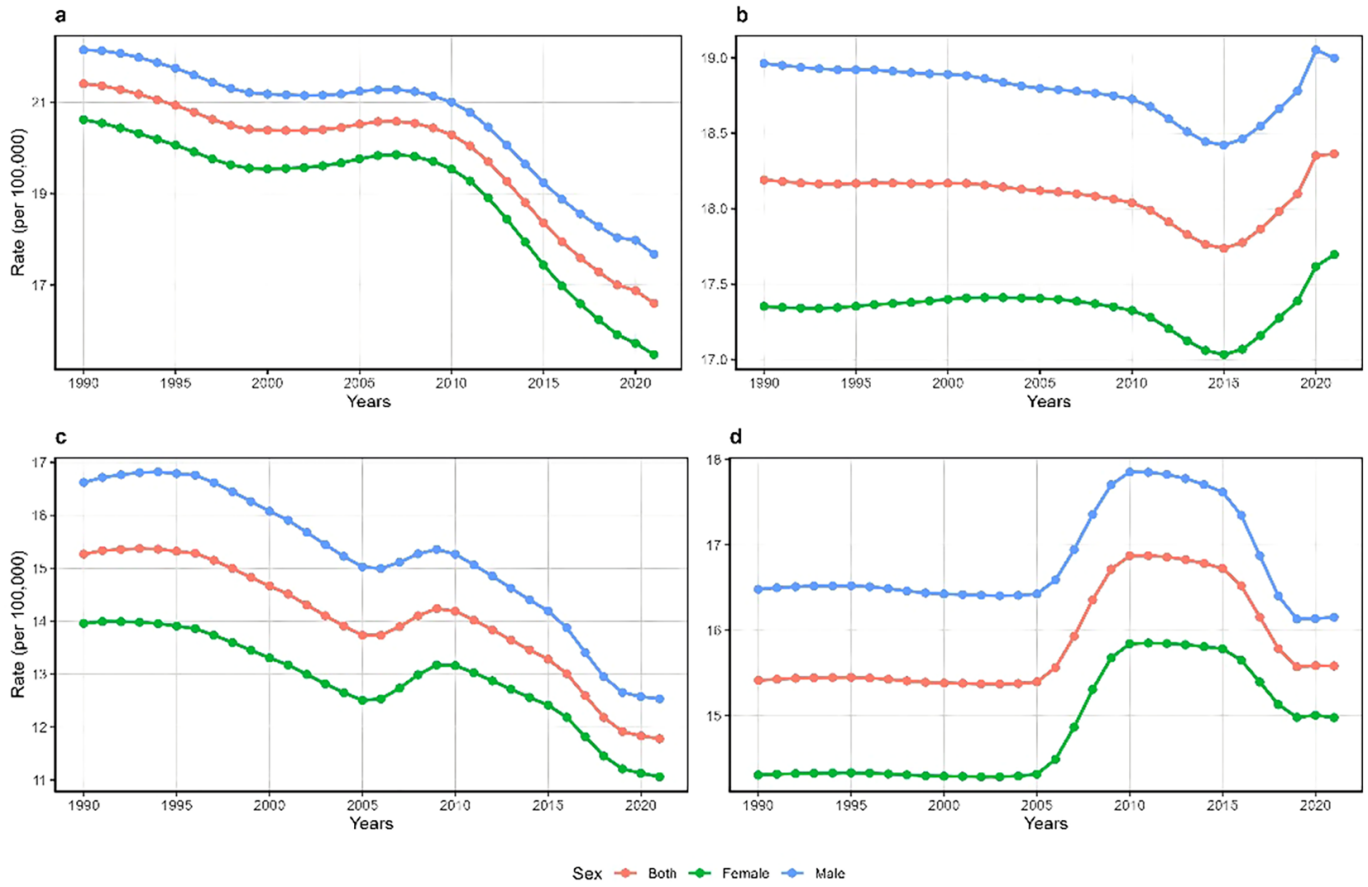
Trends in the crude incidence rate (CIR) and age-standardized incidence rate (ASIR) of schizophrenia in China [CIR: **(a)**; ASIR: **(b)**] and Japan [CIR: **(c)**; ASIR: **(d)**] from 1990 to 2021.

**Figure 1** illustrates the trajectory of the crude incidence rate (CIR) versus age-standardized incidence rates (ASIR) of schizophrenia by country and gender. China consistently demonstrated significantly higher CIR and ASIR values compared to Japan throughout the study period, irrespective of gender. Between 1990 and 2021, the CIR of schizophrenia exhibited a substantial decline in both countries: from 21.4 to 16.6 (a 22.4% decrease) in China and from 15.3 to 11.8 (a 22.9% decrease) in Japan. However, the ASIR followed distinctly different patterns. In Japan, the ASIR remained stable until 2005, then increased sharply from 15.4 to 16.9 (a 7.0% increase between 2005 and 2010, followed by a decline from 2011 to 2019, before stabilizing after 2019. In China, the ASIR showed a modest downward trend between 1990 and 2010, followed by a more pronounced decline between 2011 and 2015. Notably, from 2015 to 2020, China’s ASIR reversed course and increased from 17.7 to 18.4 (a 4.0% increase). It is noteworthy that across all the time periods, the ASIR was consistently higher in males than in females in both countries.

### 3.2 Local drift with net drift of schizophrenia incidence in China and Japan

**Figure 2:**
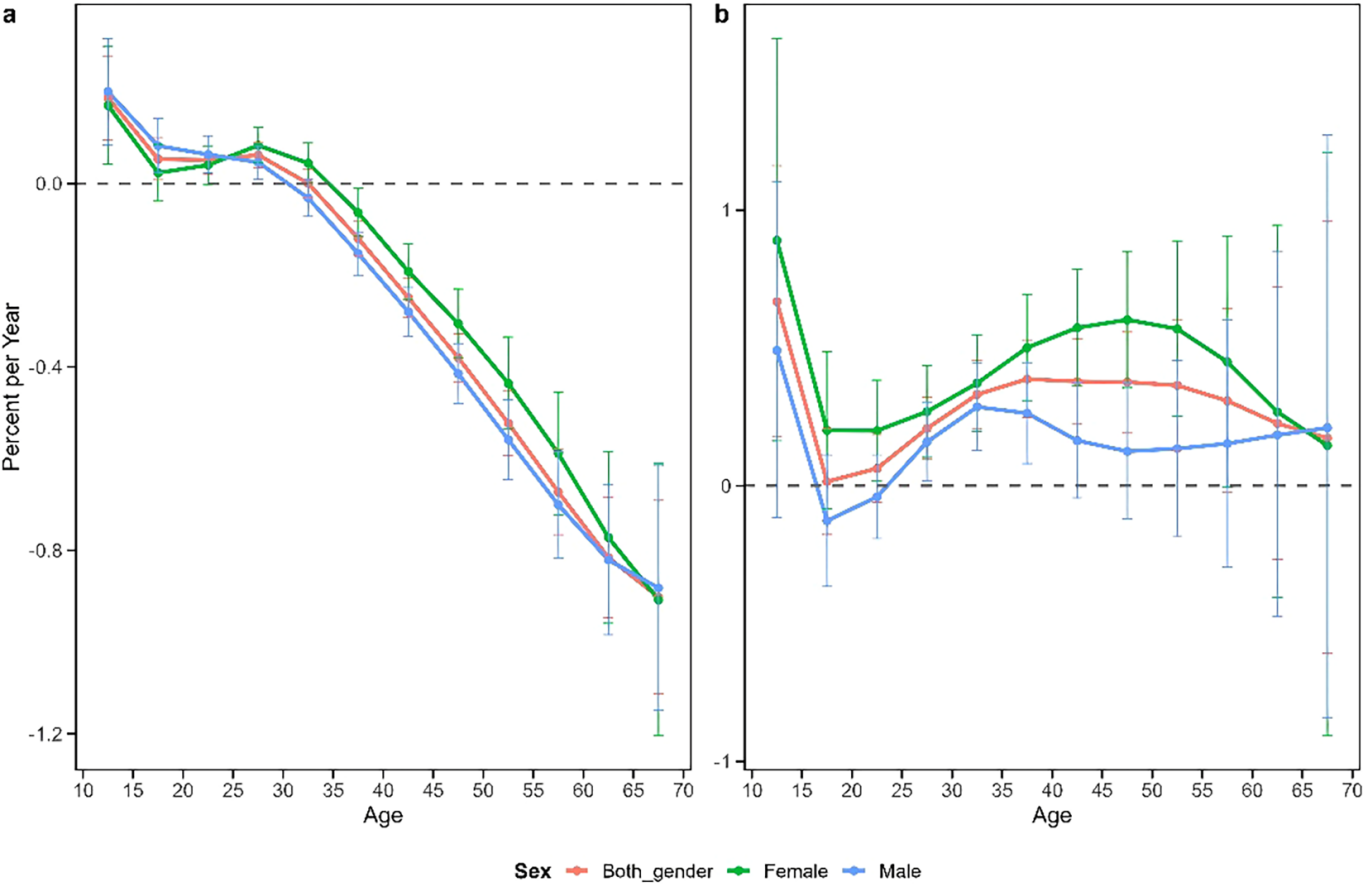
Local drift and net drift in the incidence of schizophrenia in China **(a)** and Japan **(b)**. Annual percentage change in schizophrenia at a specific age (local drift) compared with the overall annual percentage change (net drift) and corresponding 95% confidence intervals.

**Figure 2** depicts the annual rate of change in overall schizophrenia incidence (net drift) and age-specific annual rates of change (local drift) in China and Japan. The results revealed contrasting patterns between the two countries. China exhibited an overall negative net drift value of -0.25% (95% CI: -0.28% to 0.23%), with sex-specific net drifts of -0.30% (95% CI: -0.31% to -0.24%) for males and -0.20% (95% CI: -0.25% to -0.17%) for females, respectively. Conversely, Japan demonstrated a positive overall net drift value of 0.30% (95% CI: 0.20%~0.41%), with net drifts of 0.20% (95% CI: 0.03%~0.30%) for males and 0.40% (95% CI: 0.29%~0.57%) for females.

The age-specific patterns of change (local drift) revealed substantial differences between China and Japan. In China, both males and females exhibited positive local drift values in the 10–25 age group and negative values in the 35–70 age group. This suggests an improvement in schizophrenia incidence among middle-aged and older adults of both sexes, contrasted with a slight increase in incidence among adolescents and young adults. By comparison, Japanese females showed positive local drift values across all age groups, while Japanese males demonstrated negative local drift only in the 15–25 age group. This indicates that schizophrenia incidence in Japan declined only among adolescent and young adult males, while increasing among males in all other age groups and among females across all age categories. All these findings were statistically significant (p<0.05).

### 3.3 Longitudinal age curves of schizophrenia incidence rates in China and Japan

**Figure 3:**
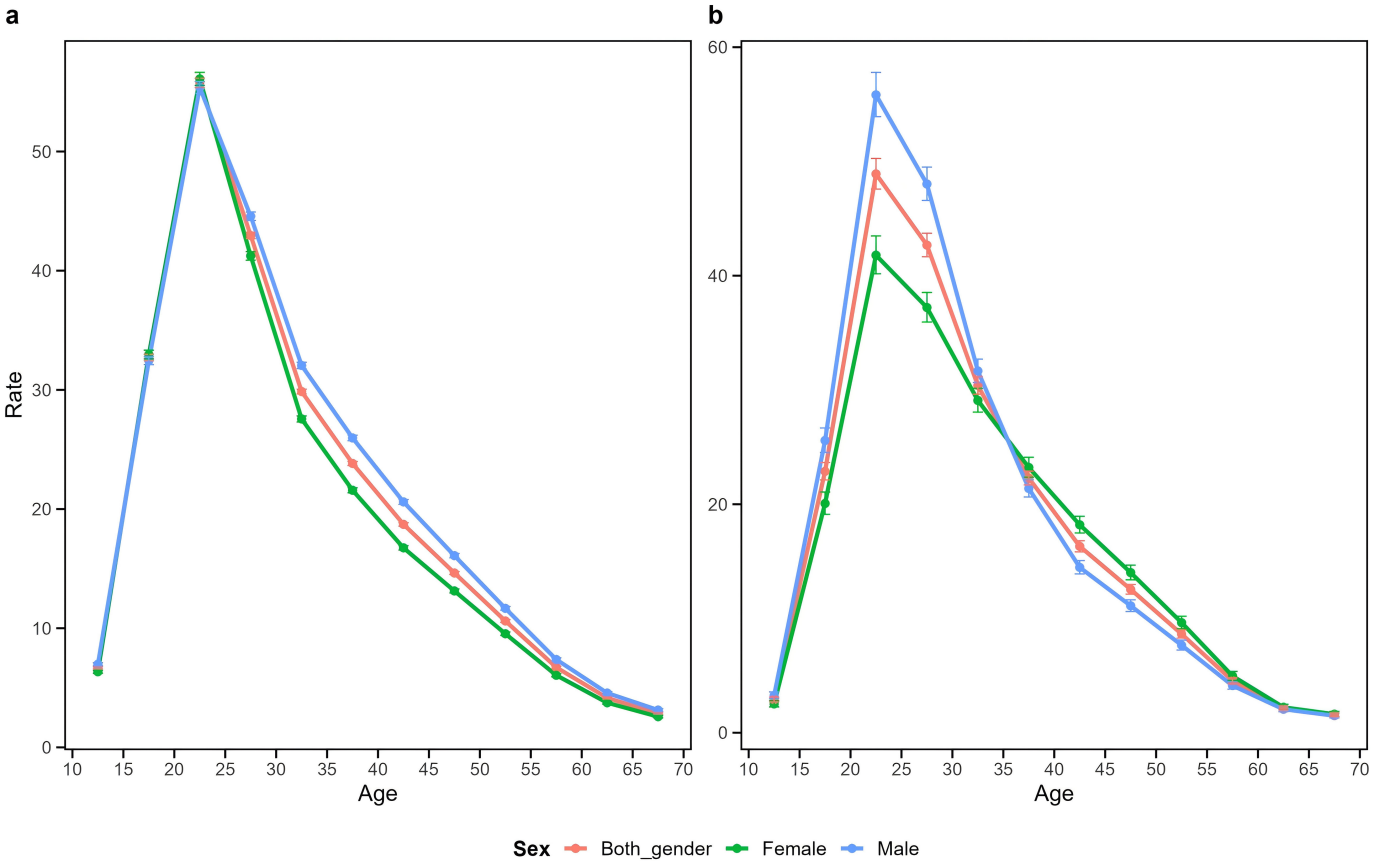
Longitudinal age curves for schizophrenia incidence in China **(a)** and Japan **(b)**. Fitted longitudinal age-specific incidence rates of schizophrenia (per 100,000 person-years) and their corresponding 95% confidence intervals, with the 35–39 age group as the reference.

**Figure 3** illustrates the age effect on schizophrenia incidence rates in China and Japan, representing the period-adjusted trend of changes with age within the same birth cohort. The results demonstrate that within the same birth cohort, schizophrenia incidence in both countries exhibited an inverted V-shaped pattern with age. Incidence rates increased rapidly from the 10–14 age group, peaked in the 20–24 age group, and subsequently declined gradually with advancing age. To further quantify the differences in incidence trends across age groups between China and Japan, we calculated the average annual percentage change (AAPC) for each age group. The results showed that the AAPC for the 10–25 age group in China was approximately 0.1-0.2%, while the AAPC for the 15–25 age group in Japan was approximately 0.2-0.3%, indicating that the rate of increase in incidence during young adulthood was slightly higher in Japan than in China. As age increased, the trends gradually diverged between the two countries: China showed a year-on-year decline in incidence after middle age (AAPC approximately -0.3% to -0.9%), while Japan maintained a mild increase (AAPC approximately +0.2% to +0.4%). These findings are consistent with the age effect curves observed in **Figure 3**, further confirming significant differences in both the direction and magnitude of incidence rate changes across different age stages between the two countries. In China, the incidence rates between males and females were largely comparable across most age groups, with males showing slightly higher rates only in the 25–30 to 50–55 age groups. Japan, however, displayed more pronounced gender differences, with significantly higher incidence rates in males compared to females in the 20–35 age group, but this pattern reversed in the 40–50 age group, where Japanese females exhibited higher incidence rates than males.

### 3.4 Period relative risk of schizophrenia incidence in China and Japan

**Figure 4:**
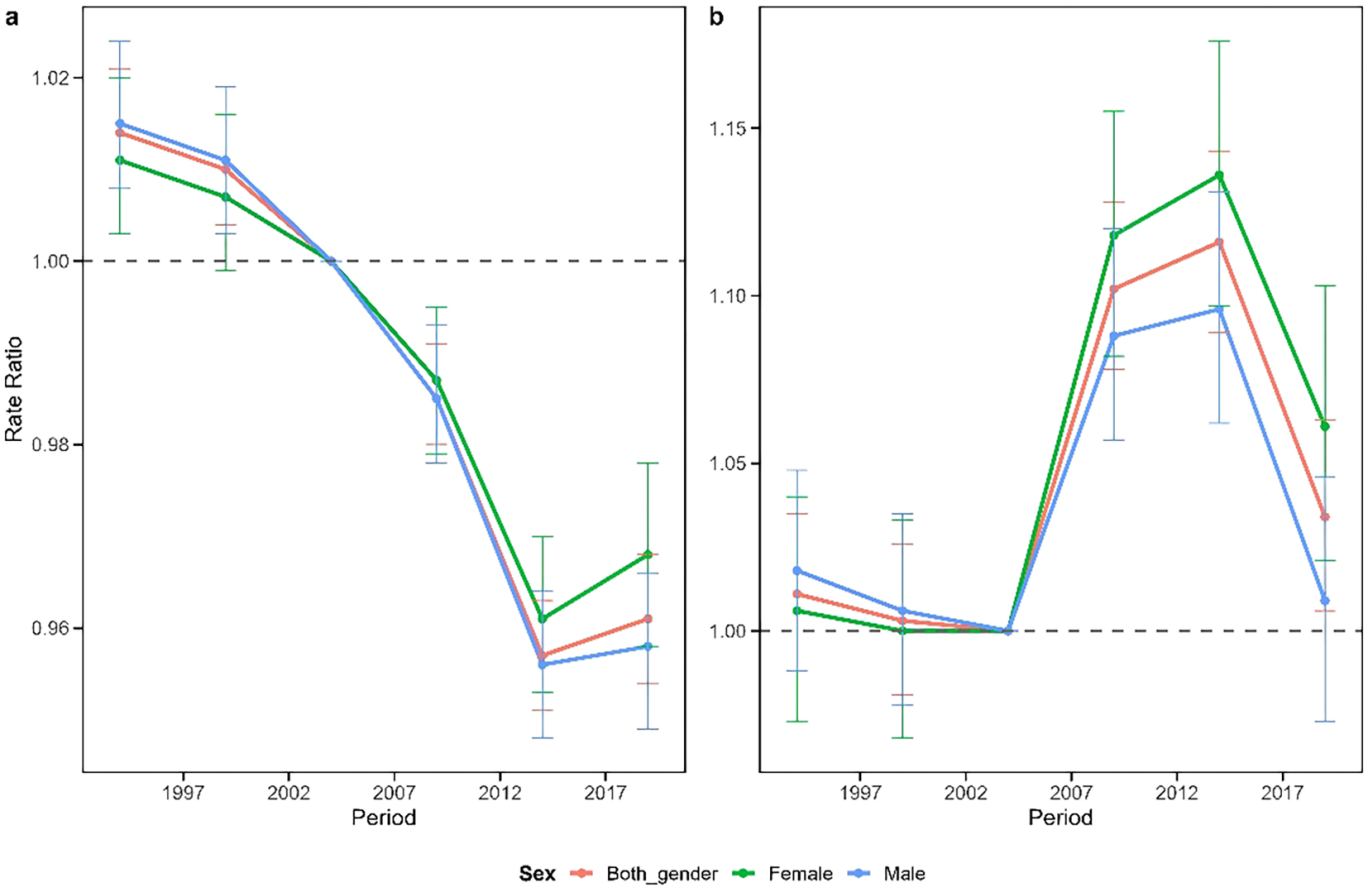
Comparison of the periodicity of schizophrenia in China **(a)** and Japan **(b)**. The periodic effects of schizophrenia incidence obtained through age-period-cohort analysis and their corresponding 95% confidence intervals, with 2002–2006 as the reference period.

**Figure 4** presents the period effect on schizophrenia incidence in China and Japan, illustrating the trend in period relative rates after adjusting for age and non-linear cohort effects. The period effect for China generally demonstrated a decreasing trend with a “V” shaped pattern. From 1992–1996 to 2012-2016, the period effect decreased consistently, followed by a slight increase from 2012–2016 onward. In contrast, Japan exhibited an inverted “N” shaped pattern in period effects. A modest decrease occurred from 1992–1996 to 2002-2006, followed by a sharp increase from 2002–2006 to 2012-2016, and subsequently a decline from 2012–2016 onward. Gender differences in period effects were minimal in China. In Japan, however, males showed slightly higher relative risk than females until 2002-2006, after which the pattern reversed, with females demonstrating significantly higher risk. Notably, the period effect values for both Japanese males and females remained greater than 1 throughout the study period. All observed period effects were statistically significant (p<0.05).

### 3.5 Cohort relative risk of schizophrenia incidence in China and Japan

**Figure 5:**
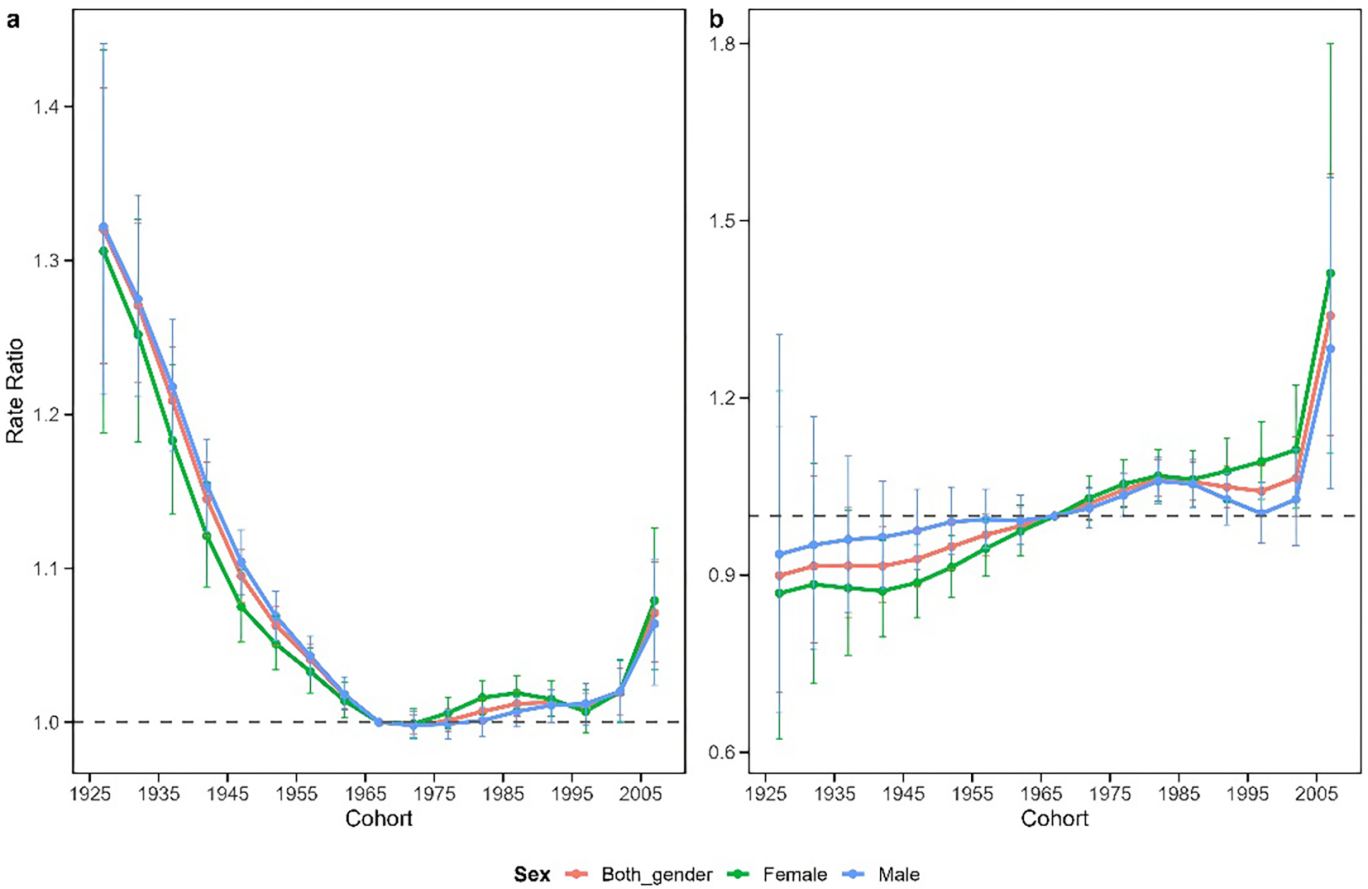
Cohort incidence ratio of mental disorders in China **(a)** and Japan **(b)**. Cohort effects of schizophrenia incidence obtained through age-period-cohort analysis and their corresponding 95% confidence intervals, with the 1965–1969 cohort as a reference.

**Figure 5** depicts the cohort effects of schizophrenia in China and Japan, representing the trend in cohort relative rates after adjusting for age and non-linear period effects. The cohort effect for China generally exhibited a “U” shaped pattern. Risk progressively decreased from the 1925–1930 birth cohort to the 1980–1985 birth cohort, with minimal risk observed in cohorts born between 1965 and 1985. However, beginning with the 1990–1995 birth cohort, the cohort risk demonstrated a concerning upward trend. The overall cohort effect in Japan displayed a distinct upward trajectory. A gradual increase in risk was observed from the 1925–1930 birth cohort to the 1980–1985 birth cohort, followed by a slight improvement in risk for cohorts born between 1980–1985 and 1995-2000. Notably, cohorts born after 2000 exhibited a sharp rise in risk, with a cumulative 48.9% increase in risk from the 1925–1930 birth cohort to the 2005–2010 birth cohort.

## 4 Discussion

This study utilized epidemiological data on schizophrenia in China and Japan from 1990 to 2021, employing the APC analysis framework to systematically assess trends in incidence rates and potential age, period, and birth cohort effects. The results indicate that the CIR in both countries has shown a declining trend, whereas the ASIR has demonstrated an increasing trend, with significant differences in their change trajectories. China’s ASIR began to rise again after 2015, while Japan’s ASIR exhibited a single-peak fluctuation and an unfavorable upward trend after 2019. The divergence between declining CIR and rebounding ASIR in China after 2015 reflects the impact of rapid population aging. The increasing proportion of elderly population mechanically reduces CIR even as age-specific rates increase in younger cohorts. The ASIR, which standardizes for age structure, more accurately captures the true increase in disease risk among recent cohorts. In both China and Japan, the incidence of schizophrenia peaked in the 20–24 age group and subsequently declined with increasing age. Although the period effects in both countries generally showed a downward trend, more recent birth cohorts displayed an unfavorable increase. The observed incidence trends are the result of combined age, period, and cohort effects, which are influenced by collective culture, social change, major historical events, and individual life trajectories.

Our analysis revealed that the age-standardized incidence of schizophrenia in both China and Japan demonstrated an overall increasing trend, consistent with global patterns (5), though China’s upward trajectory began earlier. This trend may be partially attributable to the COVID-19 pandemic through multiple mechanisms. High-dose steroid treatments commonly used to modulate inflammatory responses in COVID-19 may increase the risk of psychiatric morbidity, including psychosis, mania, delirium, and mood disturbances (12). Additionally, the medium- and long-term social consequences of the pandemic, including social isolation, unemployment, homelessness, relationship dissolution, domestic violence, and deteriorating physical health, may have exacerbated mental health burdens through social determinants of health. Furthermore, epidemics tend to intensify social inequities, further affecting associated risk factors for psychiatric disorders (13). Rapid urbanization may also contribute to fluctuations in schizophrenia incidence trends. China’s urban population has expanded nearly fivefold over four decades, and while urbanization creates opportunities for improved healthcare access, it simultaneously introduces significant risk factors associated with schizophrenia, including migration stressors, crowded living conditions, high-pressure work environments, and health security inequalities (14). Although Japan completed its urbanization process decades ago, the country now faces challenges from population aging and urban hollowing, leading to population decline, economic contraction, and weakening social support systems in urban centers and small-to-medium-sized cities. These changes may increase residents’ social isolation, reduce access to mental health resources, and elevate stress levels, potentially increasing schizophrenia risk (15).

Consistent with previous research, our findings showed that age-standardized incidence rates were consistently higher in males than females in both China and Japan (16). This gender disparity can be explained by several hypotheses. The hormonal hypothesis suggests that estrogens, particularly 17-β-estradiol, promote neuronal growth, myelin formation, synaptic density, and neuroplasticity, potentially conferring protective effects against schizophrenia in females (17). The sex chromosome hypothesis proposes that sex chromosomes influence neurodevelopment and sex-specific cognitive functions, noting that individuals with abnormal X or Y chromosomes numbers typically exhibit higher rates of movement and psychiatric disorders (18). Psychosocial factors also paly a role, males typically experience greater social and occupational stressors and tend to face more severe psychological stress during adolescence and early adulthood, partly due to their greater propensity to adopt aggressive stress-response behaviors (19). Important social structural differences also exist between the countries: while China exhibits some gender-based stressors, its overall social structure is relatively homogeneous, whereas Japan has highly polarized gender roles, with males bearing primary responsibility for education and employment, while females navigate family expectations and marriage-oriented cultural pressures (20).

The age effect typically reflects physiological differences that influence susceptibility to disease across different age groups. With regard to the age effects on schizophrenia incidence trends in China and Japan, we observed similar patterns: schizophrenia incidence increased in the 10–14 age group, peaked in the 20–24 age group, and then gradually declined with age, which is consistent with previous research findings [15]. From a biological perspective, schizophrenia is considered a neurodevelopmental disorder whose onset is associated with disruptions in the brain’s normal developmental processes. Specifically, adolescence represents a peak period for synaptic pruning, and excessive pruning may lead to abnormal connections between the prefrontal cortex and the limbic system, resulting in cognitive deficits. This, in turn, disrupts the brain’s excitatory-inhibitory balance by impairing glutamatergic neuronal function, thereby increasing the risk of schizophrenia onset [32]. From an individual perspective, adolescence is a period of intense social change. Adolescents are undergoing processes of individualization and increasing autonomy, while peer and romantic relationships become more hierarchical and complex. These factors exert significant influences on adolescents’ emotions, decision-making, risk-taking behaviors, and substance use. Additionally, prolonged exposure to social stress can trigger negative self-evaluation and social withdrawal, intensifying feelings of loneliness—which reach their peak during adolescence—and increasing the risk of mental illness [34]. From a social role perspective, adolescence is a transitional phase between childhood and adulthood, characterized by increasing academic demands, which are often regarded as the most significant source of stress for adolescents. On one hand, Confucian culture emphasizes the importance of education, viewing it as a key means to enhance personal cultivation, achieve social harmony, and govern the nation, as well as an important channel for self-realization. On the other hand, Confucian thought emphasizes filial piety, family ethics, and respect for teachers, which ties adolescents’ learning to collectivist values. Therefore, adolescents’ academic pressure often stems not only from their intrinsic desire for knowledge but also from their commitment and sense of responsibility toward family, society, and even cultural traditions, forming a deeply rooted psychological pressure system [35]. As a transitional phase toward adulthood, adolescence is a critical period for transitioning from a dependent “child role” to an autonomous “adult role,” accompanied by the reconstruction of multiple social roles. This requires adolescents to integrate fragmented social roles (child/student/peer) into a coherent self-identity; otherwise, they may fall into an identity crisis, thereby increasing the likelihood of mental disorder onset [36]. Previous studies have demonstrated that adolescents exhibit a heightened sensitivity to adversity and possess relatively lower levels of psychological resilience compared to middle-aged and older adults [37]. Consequently, they are particularly vulnerable to psychological distress, including anxiety and depression, when confronted with pressures related to academics, interpersonal relationships, and self-identity formation. Older adults, in contrast, demonstrate greater proficiency in regulating negative emotions, rationally evaluating situations, and drawing upon accumulated life experiences, resulting in significantly lower overall anxiety levels than their younger counterparts.

Period effects in our analysis reveal distinct patterns between the two countries. China’s period effect for schizophrenia incidence has shown a downward trend, falling below 1 since 2004, indicating decreased overall risk relative to earlier periods. This improvement may be partially explained by China’s successful economic reforms, which have reduced poverty and alleviated mental illness-associated stressors such as economic insecurity and unemployment. Technological advances accompanying economic development have also facilitated better recognition and management of schizophrenia (21). Concurrently, the Chinese government has implemented effective policy measures, including an eight-year mental health plan in 2002, inclusion of most new psychotropic medications in basic medical insurance in 2005, and most significantly, the “686” program in 2004, which enhanced community-based prevention and management of severe mental illnesses, thereby reducing schizophrenia risk (22). In contrast, Japan’s period effect for schizophrenia displayed considerable variability but consistently remained above 1, potentially reflecting economic fluctuations and large-scale disasters. The 1997 Asian financial crisis resulted in widespread joblessness and economic hardship, leading to severe psychological problems and elevated suicide rates (23). A positive development occurred in 2002 when the Japanese Association of Psychiatry and Neurology changed the terminology for schizophrenia from “Seishin Bunretsu Byo” (meaning “split-mind disease”) to “Togo Shitcho Sho” (meaning “integration disorder”), reducing stigma and encouraging treatment-seeking (24). However, Japan’s geographic vulnerability to natural disasters has contributed to mental health challenges, as evidenced by the 2011 Tokyo earthquake and tsunami,which caused significant loss of life and property and triggered serious mental health problems (25). The subsequent Fukushima nuclear power plant accident and radioactive material release generated substantial social anxiety (26). The Japanese government has implemented proactive mental health initiatives: in 2013, mental disorders were designated as a national health priority, the fifth most important disease category after cancer, stroke, acute myocardial infarction, and diabetes. Additionally, the Regional Healthcare Strategic Plan required local governments to assess mental health service availability, identify actions to address local needs, allocate resources, and evaluate progress (27), positively impacting national mental health outcomes.

Cohort effects in our study showed distinctive patterns between the two countries. In China, decreasing schizophrenia incidence in older birth cohorts (born before 1972) may reflect the government’s sustained efforts im mental health since the 1950s, including policies strengthening continuous management and intervention for mentally ill patients, improving mental health services, and promoting the rehabilitation and social integration (28). Beyond public health initiatives, improved social stability resulting from economic development, political relations, and enhanced international status likely influenced the significant cohort effect. China’s economic reforms since 1978 have generated sustained growth, improving living standards, facilitating mental health service expansion, and reducing pverty-related psychological distress (21). Additionally, China’s increasing stable political environment has reduced social stress and enhanced psychological security, creating conductive conditions for a healthier psychological environment (29). Conversely, Japan’s older birth cohort (born before 1975) exhibited low schizophrenia incidence levels, reflecting the post-war period of rapid economic growth characterized by improved living standards, relative social stability, and enhanced social support system. Since 1950, the Japanese government has implemented various mental health policies, including enacting the Mental Health Law, establishing the National Center for Mental Health, and introducing new therapeutic medications (30). However, Japan’s economic trajectory shifted dramatically in the late 1980s with the emergence of serious real estate and stock market bubbles, leading to economic stagnation, the “lost two decades”, characterized by mass layoffs, unemployment declining incomes, and intensifying mental health challenges (31). Since the turn of the century, rapid technological development, particularly the proliferation of smartphones and social media, has significant impacted younger generation’s lifestyles. Prolonged Internet and social media use may contribute to sleep disorders, social isolation, and mental health problems (32). Additionally, industrialization-related environmental pollution, including air pollution and noise pollution, can adversely affect population mental health (33). These factors may partially explain the increasing schizophrenia risk observed in recent birth cohorts in both China and Japan.

This study has several limitations. First, the schizophrenia incidence data in GBD 2021 primarily rely on secondary information from existing national disease registries. When population registration systems are incomplete or reporting is delayed, the estimation model often depends on prior assumptions or smoothed estimates from adjacent age groups, resulting in relatively wide Uis(uncertainty intervals,95 % UIs). This may lead to an underestimation of uncertainty in the estimation of net drift and local drift. Second, due to China’s vast territory and high level of internal migration, medical records for rural and mobile populations are often difficult to capture comprehensively. Systematic underreporting or reporting bias may therefore result in an underestimation of the overall incidence rate. Additionally, there are differences between China and Japan in terms of case registration systems, diagnostic criteria (based on different versions of the ICD), and the accessibility of mental health services. Although these discrepancies are partially adjusted for using the GBD Bayesian regression model, they may still influence the findings. Third, this study employed aggregated population-level data and applied the age-period-cohort (APC) model. Due to the potential for ecological fallacy, conclusions drawn at the population level may not be generalizable to individual-level patterns. Therefore, future individual-level studies are needed to support the findings of this research (Ferrari et al., 2021). Fourth, this study only used relevant data from China and Japan in the 2021 GBD for analysis, and did not include other East Asian countries or regions (such as South Korea, Taiwan, Singapore, etc.) for comparison. Therefore, the research findings may not comprehensively capture the overall trend of schizophrenia incidence across the entire East Asian region, and the generalizability of these results remains somewhat constrained. Future studies could extend the scope by incorporating a more diverse set of East Asian countries under a consistent methodological framework to more accurately evaluate the region’s overall disease burden pattern. Finally, while this study used the APC model to examine incidence trends and drew inferences based on existing literature, this approach lacks the capacity to quantitatively assess the impact of specific socioeconomic or cultural factors on the observed trends. Future research should incorporate individual-level longitudinal cohort or multicenter clinical data and introduce macro-level covariates—such as income inequality, educational attainment, and internal migration rates—within the APC framework to validate the robustness of our findings and quantify the contributions of key influencing factors.

Based on our findings, we propose targeted recommendations to address the disease burden of schizophrenia in China and Japan. China should prioritize strengthening mental health support for adolescents and young adults by implementing regular mental health screenings and counseling services in schools, and integrating social and emotional learning into the national curriculum. Workplace stress management should be enforced through regulations that limit excessive working hours and incorporate mental health components into employee assistance programs. At the same time, psychological services within the community-level health system should be expanded, and remote counseling as well as mobile platforms should be utilized to facilitate early intervention and provide cost-effective support. Japan’s policy framework should focus on adolescents and the elderly, and enhance cross-sectoral strategies for the prevention of suicide and severe mental disorders. Cultural stigma should be addressed through targeted public education initiatives, while families and communities should be actively encouraged to participate in mental health support efforts. Culturally responsive psychological services should also be expanded, such as by integrating local volunteer networks with traditional community-based support mechanisms. In response to the prevalent culture of long working hours and persistent gender inequality, both countries should advance key workplace reforms, including legislation to limit overtime, the promotion of flexible working schedules, and the implementation of equal pay policies. Specialized physical and psychological support programs should also be established for female employees. Furthermore, it is recommended that China and Japan regularly exchange data on mental health outcomes and intervention effectiveness, jointly develop culturally appropriate best practices tailored to East Asian contexts, and continuously refine the regional mental health service system.

## 5 Conclusion

This study presents the first comprehensive comparative analysis of long-term trends in schizophrenia incidence between China and Japan from 1990 to 2021, utilizing age-period-cohort methodology to disentangle the independent effects of these temporal factors. Our findings reveal that despite various mental health initiatives in both countries, schizophrenia incidence has not declined over the past three decades and has exhibited concerning upward trends in recent years across both nations, irrespective of gender. Consistently across both countries, males and youth populations demonstrated the highest risk of schizophrenia, with peak incidence occurring in the 20–24 age group. While China’s overall schizophrenia incidence remains substantially higher than Japan’s, our period effect analysis indicates that Japan has experienced more significant fluctuations in recent years. Most notably, both countries show alarming increases in schizophrenia risk among younger birth cohorts, a phenomenon potentially attributable to rapid technological transformation, changing social environments, and increasing environmental pollution. These finding underscore a critical public health concern: in the midst of policy focus on aging populations, the deteriorating mental health of younger generations demands urgent attention. Policymakers in China and Japan must recognize the necessity of developing country-specific and age-targeted mental health strategies that account for their unique socioeconomic contexts, cultural factors, and healthcare infrastructures. By leveraging existing healthcare systems and social services while acknowledging theses distinctive national characteristics, both countries can implement more effective and tailored approaches to reduce the burden of schizophrenia and improve mental health outcomes across diverse population segments. This study highlights the value of comparative epidemiological analysis in informing evidence-based mental health policy and emphasizes the need for continued surveillance of schizophrenia trends, particularly among vulnerable youth populations in East Asian countries experiencing rapid social and economic transformation.

## Data Availability

Publicly available datasets were analyzed in this study. This data can be found here: http://ghdx.healthdata.org/gbd-results-tool.
